# Incidence and treatment of adult femoral fractures with osteogenesis imperfecta: An analysis of a center of 72 patients in Taiwan

**DOI:** 10.7150/ijms.53286

**Published:** 2021-01-14

**Authors:** Chung-Lin Lee, Shih-Chia Liu, Chen-Yu Yang, Chih-Kuang Chuang, Hsiang-Yu Lin, Shuan-Pei Lin

**Affiliations:** 1 Department of Pediatrics, MacKay Memorial Hospital, Hsinchu, Taiwan.; 2 Institute of Clinical Medicine, National Yang-Ming University, Taipei, Taiwan.; 3 Department of Orthopedics, MacKay Memorial Hospital, Taipei, Taiwan.; 4 Department of Medicine, MacKay Medical College, New Taipei City, Taiwan.; 5 Division of Genetics and Metabolism, Department of Medical Research, MacKay Memorial Hospital, Taipei, Taiwan.; 6 College of Medicine, Fu-Jen Catholic University, Taipei, Taiwan.; 7 Department of Pediatrics, MacKay Memorial Hospital, Taipei, Taiwan.; 8 MacKay Junior College of Medicine, Nursing and Management, Taipei, Taiwan.; 9 Department of Medical Research, China Medical University Hospital, China Medical University, Taichung, Taiwan.; 10 Department of Rare Disease Center, MacKay Memorial Hospital, Taipei, Taiwan.; 11 Department of Infant and Child Care, National Taipei University of Nursing and Health Sciences, Taipei, Taiwan.

**Keywords:** adult, femoral fracture, non-union, osteogenesis imperfect, Taiwan

## Abstract

**Background:** Osteogenesis imperfecta (OI) is a rare disease characterized by increased bone fragility and susceptibility for fractures. Only few studies have compared the management for femoral fractures in children with OI. Nevertheless, no cohort studies have described the treatment for femoral fractures in adults with OI in Taiwan. This study aimed to investigate and compare the incidence of union and non-union femoral fractures and the best treatment options to avoid non-union fractures.

**Methods:** We enrolled 72 patients with OI who were older than 18 years at MacKay Memorial Hospital between January 2010 and December 2018. Femoral fracture incidence, non-union rate, and treatment modality were analyzed.

**Results:** Of 72 patients with OI, 11 patients had femoral fractures and 4 patients of them had >1 femoral fracture. The incidence for all types of femoral fractures was 651 fractures per 100,000 person-years annually. In 15 total fractures, 4 fractures resulted in non-union, and patients with type 4 OI mostly had shaft fractures. The best outcomes for non-union shaft fracture is achieved by surgical treatment.

**Conclusion:** Adults with OI tended to develop femoral fractures and non-unions. Adults with type 4 OI were particularly at high risk for non-unions in shaft fractures with conservative treatment.

## Introduction

Osteogenesis imperfecta (OI) is a rare connective tissue disease characterized by increased bone fragility and is also known as brittle bone disease. In addition, 90% of patients with OI have mutations in the *COL1A1* or *COL1A2* gene, which encodes for alpha-1 and alpha-2 chains in type 1 collagen 1. Fractures and skeletal deformation are easily seen in patients with OI because bone tissue is mainly composed of type 1 collagen 2. OI is also characterized by blue sclerae, dentinogenesis imperfecta, hyperlaxity, hearing loss, and short stature 3-5.

According to clinical variability and pattern of inheritance, Sillence classification has four OI types (types 1-4) (Table 1) 6. Additional types have been added later, and type 5 is accepted worldwide. Type 1 is the mildest form of OI, and patients usually have blue sclerae and frequently have dentinogenesis imperfecta. Type 2 is a perinatal lethal form. Type 3 is the most severe (non-fatal) form of OI with multiple fractures, progressive deformity, and short stature. Patients are often wheelchair bound. Type 4 has a variable degree of deformity with normal sclerae. Patients with type 4 OI have increased fracture risk. The most frequent type in OI is type 1. Type 5 is characterized by mild to severe weak bones. Progressive calcifications of the interosseous membrane of forearm and lower leg are observed. Some patients also develop hyperplastic callus formation after fractures 3. Bone fragility and fracture risk increase in the following order: Type 1 < Types 4, 5 < Type 3 < Type 2 7.

Although femoral fractures are common in patients with OI, no studies have described the fracture and non-union incidence in adults due to the rarity of this disease 8-10. The non-union rate in adults with OI is expected to be higher than that in non-adults with OI. Surgical treatment is more challenging due to anatomical and bone-related abnormalities 9, 11-13. This study aimed to investigate the incidence of femoral fractures and non-unions in adults with OI and to review our experience on the best possible treatment for non-union fracture.

## Methods

### Participants

This retrospective study was conducted in the OI expert clinic for adults at MacKay Memorial Hospital, Taipei, Taiwan. Patients with OI with a femoral fracture at age 18 years or older were retrieved from January 2010 to December 2019. We excluded patients with an osseous primary tumor, metastatic disease, or prednisone use due to an increased fracture risk. Radiographs were taken due to recent bone fracture or localized pain, suggesting possible fracture. All patients (n=72) were confirmed the diagnoses by molecular analysis. Molecular diagnosis was performed using a combination of Sanger sequencing and targeted resequencing directed to scan the entire coding sequence. The publicly available databases DGV (Database of Genomic Variants), DECIPHER (Database of Chromosomal Imbalance and Phenotype in Humans Using Ensembl Resources), OMIM (Online Mendelian Inheritance in Man), PubMed, ClinVar and the UCSC Genome Browser were used to compare the present findings with previous reports and evaluate the morbidity of the candidate gene.

**Figure 1:**
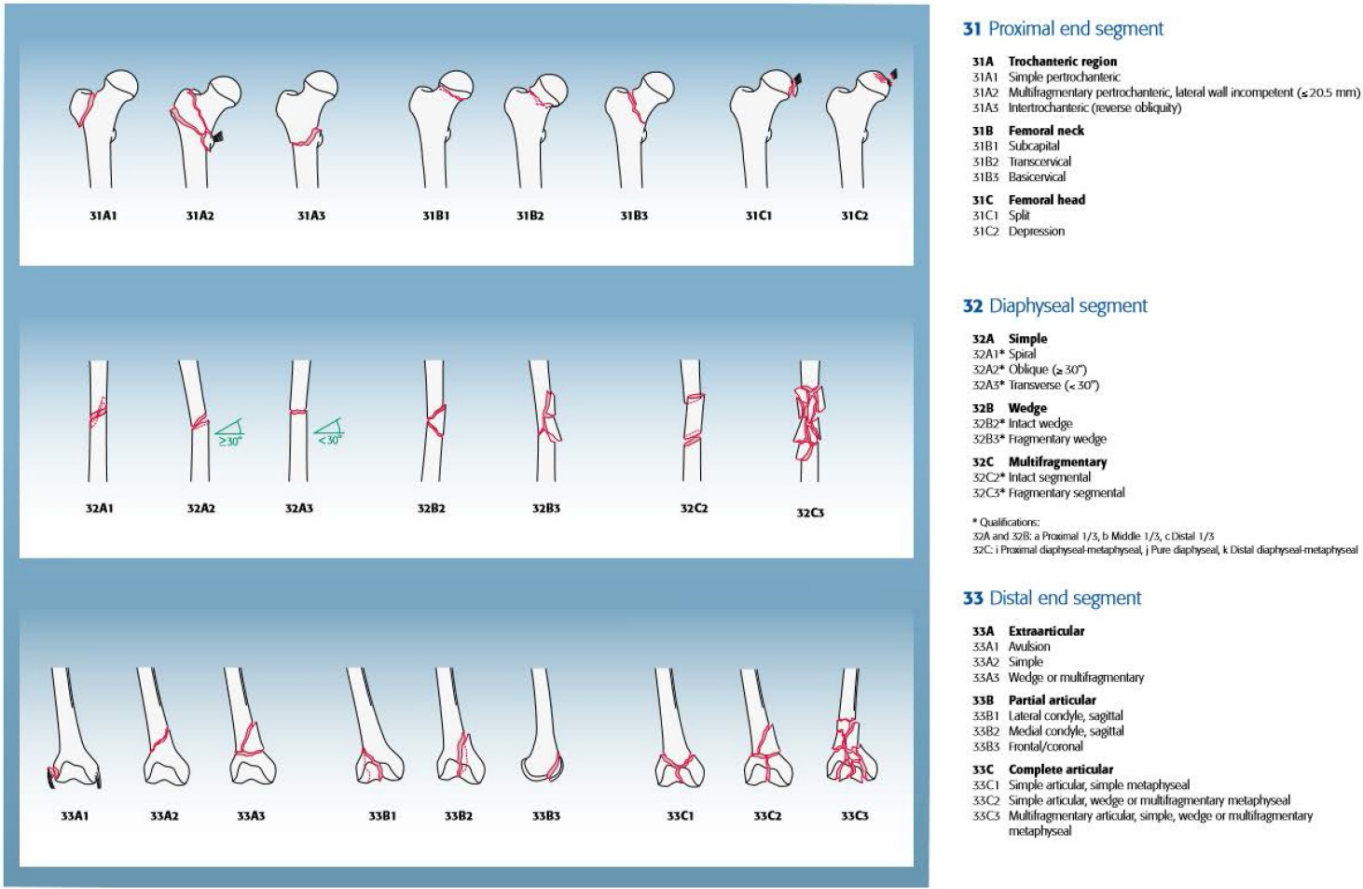
AO/OTA fracture and dislocation classification of femur (Meinberg EG, Agel J, Roberts CS, et al., 2018).

Besides demographic characteristics, we recorded the type of OI according to the Sillence classification. Femoral fractures were classified according to the AO/OTA Fracture and Dislocation Classification of Femoral Fractures (proximal, shaft, and distal, Figure 1) 14, type of treatment (intramedullary nailing [IN], plate fixation [PF], conservative) and outcome (union, non-union). Radiographic follow-up was used to determine union, defined as the presence of bridging callus in at least three of four cortices, which were evaluated on radiographs in two transverse levels 15. Non-union was defined as non-radiographic changes to union or the absence of bridging callus of two or more cortices, which were evaluated on radiographs in two transverse levels, for at least 6 months after surgical or conservative treatment 16-17.

### Statistical methods

A descriptive study was conducted because this study contains a relatively small number of subjects. Descriptive statistics were used to analyze the results using SPSS version 22.0 (SPSS, Inc., Chicago, IL). Categorical variables were expressed as percentage, and metric variables as mean and standard deviation.

## Results

**Figure 2:**
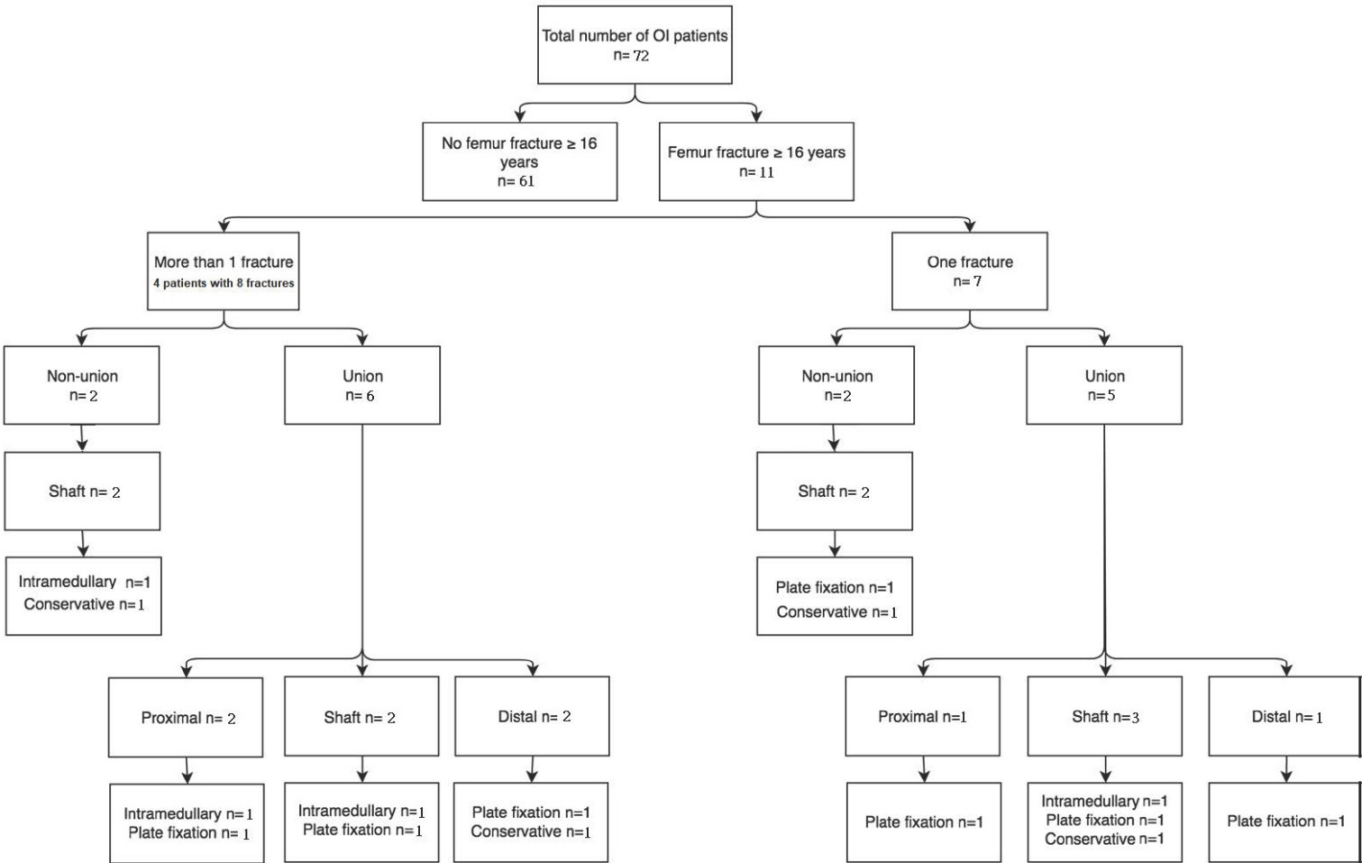
Flow diagram of fracture pattern and treatment.

Table 2 shows the demographic characteristics of study subjects with OI. Subdivision of patients was based on the history of femoral fracture and, subsequently, history of non-union. Eleven (15.3%) patients had a femoral fracture (Figure 2). Four of these patients had more than one femoral fracture (total of eight fractures), resulting in 15 femoral fractures.

### Influence of OI type on healing rate

Patients with OI types 3 and 4 were prone to femoral fractures; three (30.0%) and four (33.3%) of these patients with OI had at least one fracture. Of all patients with type 1 OI, four (8.0%) had one femoral fracture, and no patient had more than one femoral fracture. Of 15 femoral fractures, four fractures resulted in non-unions. All four non-unions were shaft fractures (Table 3; Figure 2).

### Influence of treatment on healing rate

**Figure 3:**
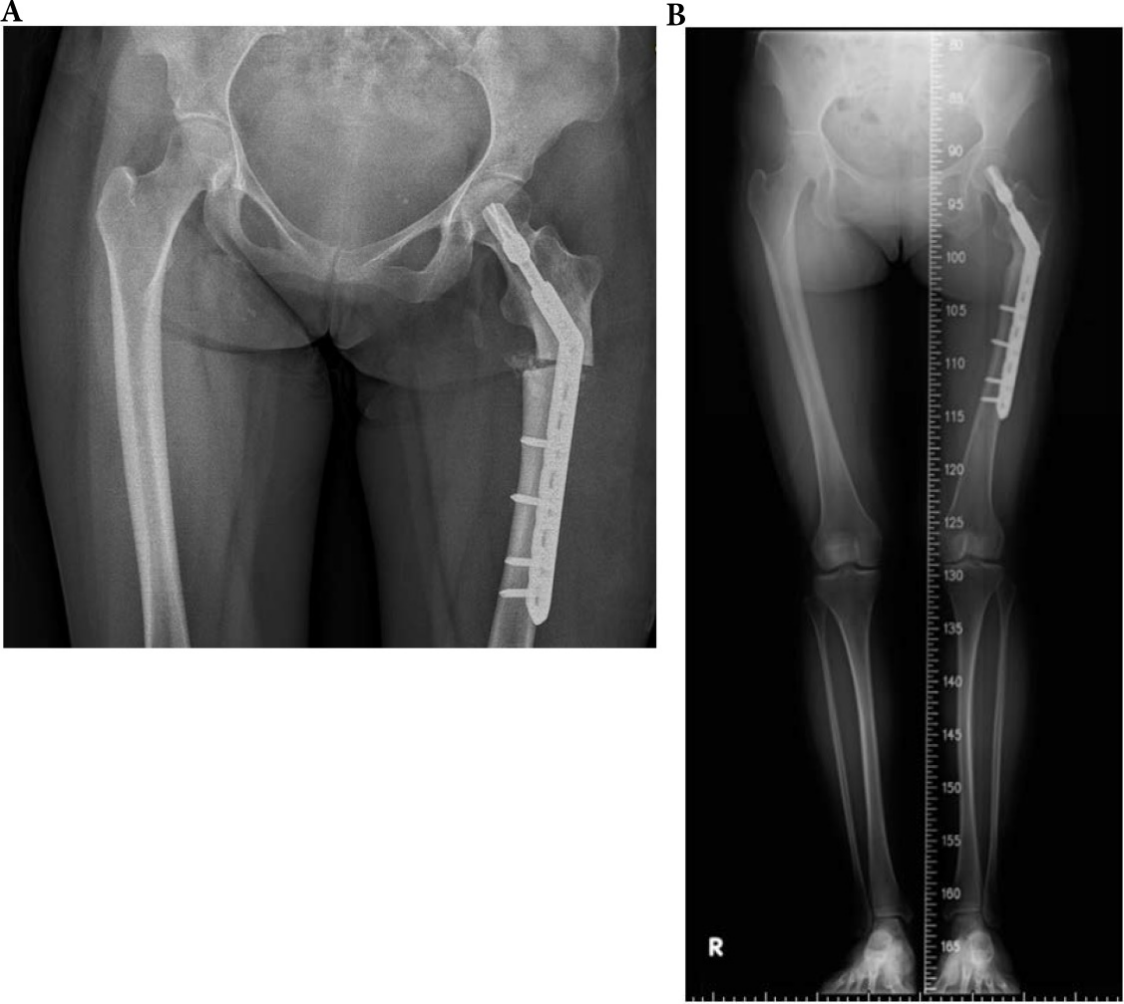
A 33-year-old type 4 OI female suffered a left shaft femur fracture with plate fixation treatment in December 2015, resulting in union. (a) Just finished operation in December 2015 (b) Follow up in August 2016.

**Figure 4:**
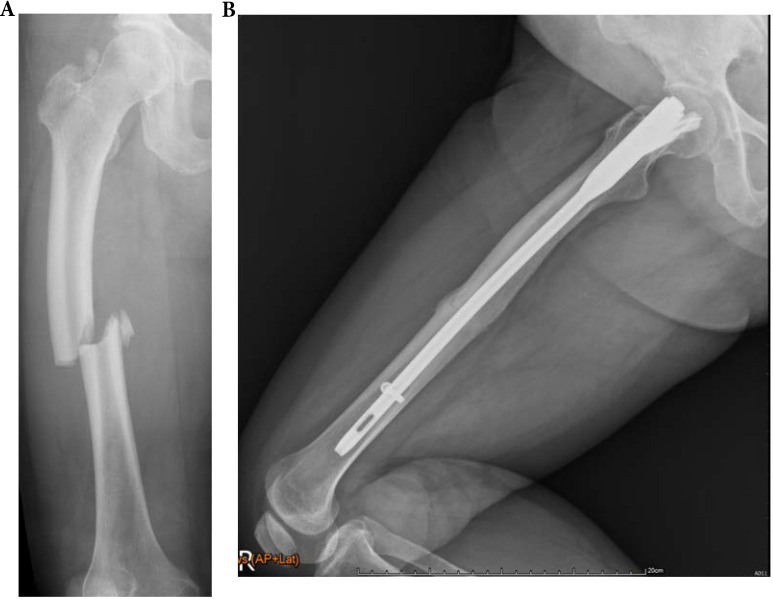
A 71-year-old female patients with type 4 OI had right midshaft femoral fracture with intramedullary nailing treatment in November 2014, which resulted in union. (a) Pre-operation fracture picture (b) Follow up in May 2016.

**Figure 5:**
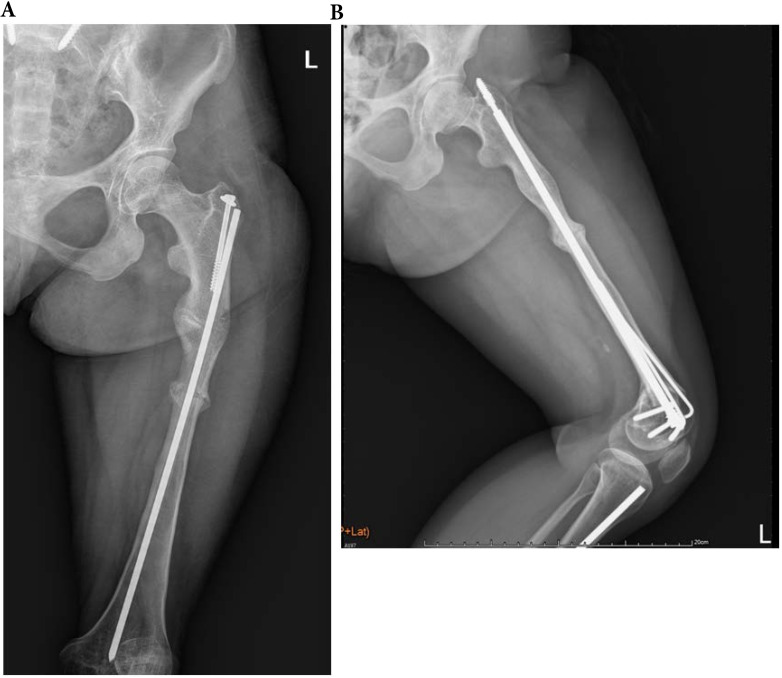
A 23-year-old female with type 4 OI had left distal femoral fracture with intramedullary nailing treatment in July 2016, which resulted in union. (a) Two weeks after operation (b) Follow up in July 2017.

**Figure 6:**
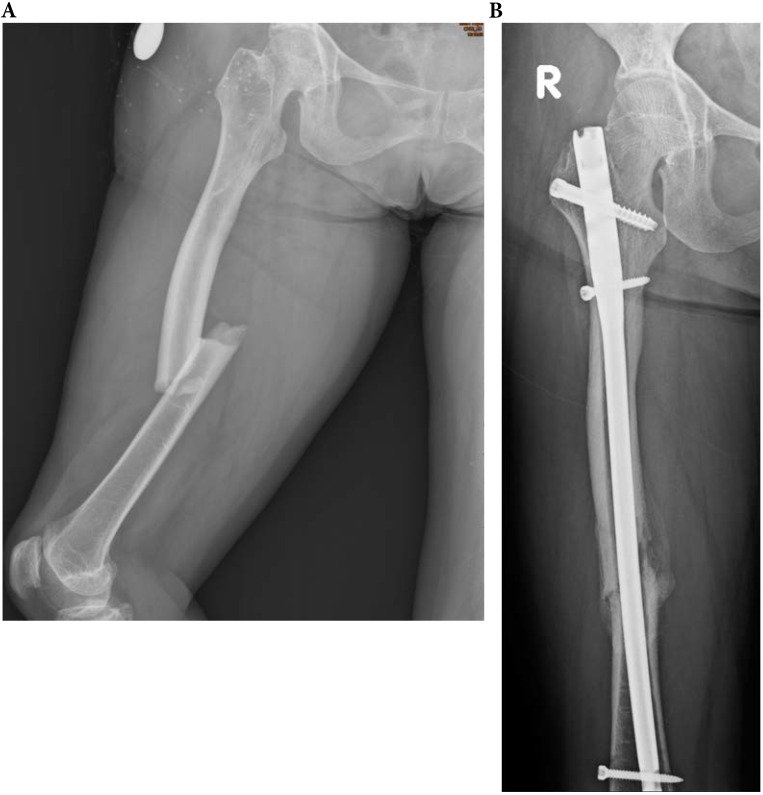
A 61-year-old type 4 OI female suffered a right midshaft femur fracture with intramedullary nailing treatment in August 2018, resulting in non-union. (a) Pre-operation fracture picture (b) Follow up in December 2019.

**Figure 7:**
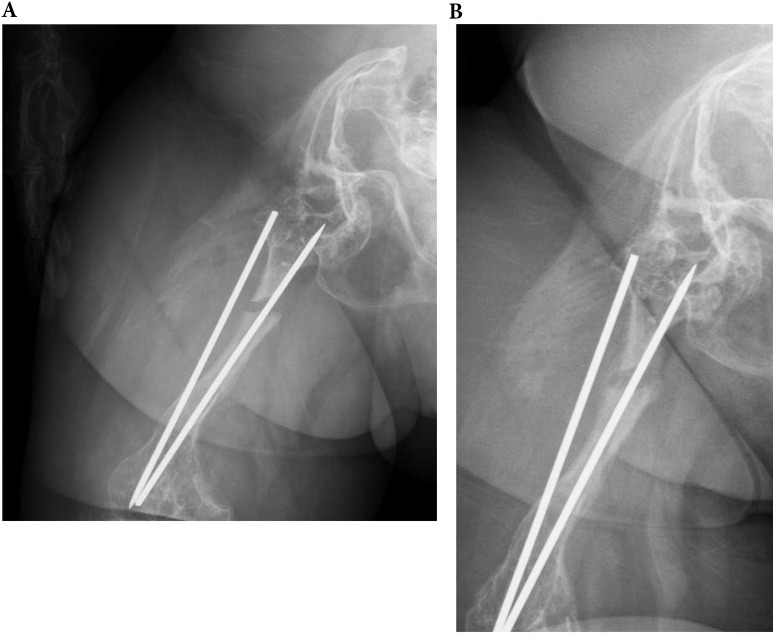
A 29-year-old type 3 OI female suffered a right shaft femur fracture with intramedullary nailing treatment in March 2012, resulting in non-union. (a) Just finished operation in March 2012 (b) Follow up in April 2013.

In OI type 4, 2 of 5 (40.0%) fractures resulted in non-union. All two patients with type 4 OI who were conservatively treated with OI had shaft fractures resulting in non-unions. Three other patients with type 4 were surgically treated for shaft fractures. All resulted in union (2 IN, 1 PF) (Figures 3, 4, 5). In patients with OI with type 1 OI, 1 of 4 (25.0%) fractures resulted in non-union (Figure 6), for type 3, 1 of 6 (16.7%) fractures resulted in non-union (Figure 7).

Overviewing all shaft fractures (n=9), intramedullary fixated fractures resulted in one non-union of three fractures (33.3%). Plate-fixated fractures resulted in one non-union of three fractures (33.3%). Conservatively treated shaft fractures resulted in two non-unions of three fractures (66.6%).

## Discussion

In this study, we reviewed 72 adult patients with OI with or without femoral fractures and unions or non-unions. Eleven patients had 15 femoral fractures (15.3%) and 4 non-unions (26.7%). Adults with type 4 OI tended to develop non-unions by conservative treatment for midshaft fractures similar to a previous study 18. For all types of femoral fractures, the incidence was 651 fractures per 100,000 person-years annually. The incidence was 355 fractures per 100,000 and 10 per 100,000 person-years annually for shaft fractures and femoral shaft fractures, respectively 19-20. These results reveal a convincing discrepancy that patients with OI have more femoral fractures than patients with non-OI.

This is the first study describing the incidence and non-union rate of femoral fractures in adults with OI in Taiwan. Some smaller studies compared outcomes between different treatments in children with OI. Chiarello et al. compared the outcomes between surgical and conservative treatments of 29 children with long bone fragility fractures. They reported a slightly lower non-union and delayed-union rates under surgical treatment 21. Enright and Noonan described femoral and tibial fractures with bone plating in four children with type 3 OI, which resulted in high complication rates 22. Agarwal and Joseph and Gamble et al. found a 15%-20% prevalence on non-union fracture in a heterogeneous group of children with OI over a 10-14-year period 23-24. However, the number of patients is small, and the results could not be extrapolated to adults, whereas it is 7.3 times higher non-union rate compared with adults with non-OI than in children with non-OI 25.

## Limitations

Although our study revealed new insights on the incidence of femoral fractures and non-union in adults with OI, it also has limitations in analyzing for non-union. There are many causative factors for non-union, including bisphosphonate use, DXA scans, smoking status, nutritional deficiency, vitamin D deficiency, mobilization status, metabolic disease, or endocrine pathology 26. We were unable to adjust these confounders due to lost data of these factors. Furthermore, 34 patients (47.2%) received bisphosphonate treatment after the diagnosis of OI in our study and all patients with femur fractures using bisphosphonate. It might cause that non-union fractures turn to union fractures even if under conservative treatment. Additionally, in our patients with femur fractures (n=11), 9 patients had femoral implants (nail or plate) before the diagnosis of OI. This may influence the number of fractures in adult life. Moreover, we also needed more patients' data to strengthen the results due to the limited number of fractures.

## Recommendations for shaft fractures

Although this study has its limitations, we could state that adults with OI have a high tendency for non-unions with conservative treatment in femoral shaft fractures. We recommend surgical treatment for shaft fractures according to our data. We suggest intramedullary nailing in the surgical treatment for femoral shaft fractures despite the fact that our study results in no definite consensus. Intramedullary nailing is the standard treatment for femoral shaft fracture in adults with non-OI. Its advantages include optimal mechanical stability, efficient load transfer, minimization of stress concentration, early mobilization of hip and knee, preservation of soft tissues, fracture hematoma, and periosteal blood supply 17, 27-29. Karadimas et al. reviewed the complications of intramedullary nails in non-OI femoral fracture. They reported a non-union rate of 1%-14.1% 30. Excluding the study by Noumi et al., which only included open fractures, the non-union rates decreased to 1%-7.6% 31-32. We could use a smaller nail in cases with anatomical abnormalities. It is also practical to use a humeral nail in cases of narrow intramedullary canal with a wedge osteotomy, if necessary. Patients' care should be personalized based on their characteristics, type of fracture, anatomical situation, and pre-existent materials. Caring for these patients with centralization and multidisciplinary work-up is needed (orthopedic surgeon, anesthetist, rehabilitation specialist, physiotherapist, occupational therapist, internist, geneticist, and radiologist).

## Conclusion

In conclusion, adult patients with OI had a high incidence of femoral fractures and non-union rates. Conservative treatment in femoral shaft fractures in adults with OI had a high tendency for non-unions, particularly for patients with type 4 OI, probably because determining the severity of fracture in patients with type 4 OI is difficult for physicians, and they would hesitate to treat patients by conservative or surgical treatment. This study provides valuable features of a unique collection of patients with OI. However, confounding factors were not analyzed, and larger cohort studies are needed to determine the best treatment for femoral fractures in adult patients with OI.

## Figures and Tables

**Table 1:** A new OI nomenclature combined with causative genes (A) Phenotypes with mild to moderate severity, (B) Progressively deforming and perinatally lethal phenotypes (Van Dijk FS, Sillence DO, 2014) (AD: autosomal dominant; AR: autosomal recessive; XL: X-linked recessive)

OI syndrome names	Type	Gene	Locus	Protein product	Inheritance
(A) Non-deforming OI with blue sclerae	1	*COL1A1*	17q21.33	Collagen alpha-1(I) chain	AD
		*COL1A2*	7q22.3	Collagen alpha-2(I) chain	AD
Common variable OI with normal sclerae	4	*COL1A1*	17q21.33	Collagen alpha-1(I) chain	AD
		*COL1A2*	7q22.3	Collagen alpha-2(I) chain	AD
		*WNT1*	12q13.12	Wingless-type MMTV integration site family, member 1	AD
		*CRTAP*	3p22.3	Cartilage-associated protein (CRTAP)	AR
		*PPIB*	15q22.31	Cyclophilin B (CyPB)	AR
		*SP7*	12q13.13	Osterix	AR
		*PLS3*	Xq23	Plastin 3	XL
OI with calcification in interosseous membranes	5	*IFITM5*	11p15.5	Interferon-induced transmembrane protein 5	AD
(B) Progressively deforming	3	*COL1A1*	17q21.33	Collagen alpha-1(I) chain	AD
		*COL1A2*	7q22.3	Collagen alpha-2(I) chain	AD
		*BMP1*	8p21.3	Bone morphogenetic protein 1	AR
		*CRTAP*	3p22.3	Cartilage-associated protein (CRTAP)	AR
		*FKBP10*	17q21.2	Peptidyl-prolyl cis-transisomerase	AR
		*LEPRE1*	1p34.2	Prolyl 3-hydroxylase 1 (P3H1)	AR
		*PLOD2*	3q24	Procollagen-lysine, 2-oxoglutarate	AR
		*PPIB*	15q22.31	5-dioxygenase 2	AR
		*SERPINF1*	17p13.3	Cyclophilin B (CyPB)	AR
		*SERPINH1*	11q13.5	Pigment-epithelium-derived factor (PEDF)	AR
		*TMEM38B*	9q31.1	Heat shock protein 47 (HSP47)	AR
		*WNT1*	12q13.12	Trimeric intracellular cation channel B (TRIC-B)	AR
		*CREB3L1*	11q11	Wingless-type MMTV integration site family, member 1	AR
				Old Astrocyte	AR
				Specifically induced substance (OASIS)	AR
Perinatally lethal OI	2	*COL1A1*	17q21.33	Collagen alpha-1(I) chain	AD
		*COL1A2*	7q22.3	Collagen alpha-2(I) chain	AD
		*CRTAP*	3p22.3	Cartilage-associated protein (CRTAP)	AR
		*LEPRE1*	1p34.2	Prolyl 3-hydroxylase 1 (P3H1)	AR
		*PPIB*	15q22.31	Cyclophilin B (CyPB)	AR

**Table 2:** Demographic characteristics of study subjects with osteogenesis imperfecta

	All patients (n = 72)	No femur fracture (n = 61)	Femur fracture (n = 11)
Age (years)	41.0 ± 15.3	40.4 ± 15.4	44.1 ± 14.7
Gender (male, %)	25 (34.7)	20 (32.8)	5 (45.5)
**OI type (n, %)**			
1	50 (69.4)	45 (73.8)	5 (45.5)
2	0 (0.0)		
3	10 (13.9)	8 (13.1)	2 (18.2)
4	12 (16.7)	8 (13.1)	4 (36.3)

Data are presented as mean ± standard deviation or number of patients and percentages of group.

**Table 3:** Patient characteristics comparing union and non-union groups in osteogenesis imperfecta, all femur fractures were included if age ≥18 years

	Union (n = 11)	Non-union (n = 4)
Age (years)	44.9±14.4	45.5±13.1
Age at fracture (years)	36.3±13.0	40.1±13.1
Gender (male, %)	6 (54.5)	1 (25.0)
**OI type (n, %)**		
1	3 (27.3)	1 (25.0)
2	0 (0.0)	0 (0.0)
3	5 (45.4)	1 (25.0)
4	3 (27.3)	2 (50.0)
**Type of fracture (n, %)**		
Proximal	3 (27.3)	0 (0.0)
Shaft	5 (45.4)	4 (100.0)
Distal	3 (27.3)	0 (0.0)
**Type of treatment (n, %)**		
Plate fixation	6 (54.5)	1 (25.0)
Intramedullary fixation	3 (27.3)	1 (25.0)
Conservative	2 (18.2)	2 (50.0)

Data are presented as mean ± standard deviation or number of patients and percentages of group.
